# Foodborne Pathogenic Vibrios: Antimicrobial Resistance

**DOI:** 10.3389/fmicb.2021.638331

**Published:** 2021-06-30

**Authors:** Dipanjan Dutta, Anupam Kaushik, Dhirendra Kumar, Satyabrata Bag

**Affiliations:** ^1^Department of Biochemistry, Panjab University, Chandigarh, India; ^2^Department of Microbiology, National Centre for Disease Control, New Delhi, India; ^3^3B BlackBio Biotech India Limited, Bhopal, India

**Keywords:** foodborne Vibrio, antimicrobial resistance, seafood, drug resistant bacteria, mobile genetic elements

## Abstract

Foodborne illness caused by pathogenic Vibrios is generally associated with the consumption of raw or undercooked seafood. Fish and other seafood can be contaminated with Vibrio species, natural inhabitants of the marine, estuarine, and freshwater environment. Pathogenic Vibrios of major public health concerns are *Vibrio cholerae*, *Vibrio parahaemolyticus*, and *Vibrio vulnificus*. Common symptoms of foodborne Vibrio infection include watery diarrhea, stomach cramping, nausea, vomiting, fever, and chills. Administration of oral or intravenous rehydration salts solution is the mainstay for the management of cholera, and antibiotics are also used to shorten the duration of diarrhea and to limit further transmission of the disease. Currently, doxycycline, azithromycin, or ciprofloxacin are commonly used for *V. cholerae*, and doxycycline or quinolone are administered for *V. parahaemolyticus*, whereas doxycycline and a third-generation cephalosporin are recommended for *V. vulnificus* as initial treatment regimen. The emergence of antimicrobial resistance (AMR) in Vibrios is increasingly common across the globe and a decrease in the effectiveness of commonly available antibiotics poses a global threat to public health. Recent progress in comparative genomic studies suggests that the genomes of the drug-resistant Vibrios harbor mobile genetic elements like plasmids, integrating conjugative elements, superintegron, transposable elements, and insertion sequences, which are the major carriers of genetic determinants encoding antimicrobial resistance. These mobile genetic elements are highly dynamic and could potentially propagate to other bacteria through horizontal gene transfer (HGT). To combat the serious threat of rising AMR, it is crucial to develop strategies for robust surveillance, use of new/novel pharmaceuticals, and prevention of antibiotic misuse.

## Introduction

Vibrio is a genus of Gram-negative bacteria under Vibrionaceae family, commonly found in aquatic environments, including marine, estuarine, and aquaculture settings. As such, they become common flora of marine life, including those consumed as seafood. So far more than 100 species of Vibrio are identified but approximately 12 species of Vibrio including *Vibrio vulnificus*, *Vibrio parahaemolyticus*, and *Vibrio cholerae* are the leading cause of food borne vibriosis in humans worldwide (Austin et al., 2005). Particularly, two Vibrio species viz. *V. vulnificus* and *V. parahaemolyticus* are the significant foodborne human pathogens, and the infections usually occur with the consumption of naturally contaminated raw, undercooked or cross- contaminated shellfish (oysters, crabs, and/or shrimp) and fish (sushi or sashimi) (Altekruse et al., 2000; Dechet et al., 2008). Highest number of seafood-associated bacterial gastroenteritis is caused by *V. parahaemolyticus* in many countries including United States and Asian countries (Liu et al., 2006; Scallan et al., 2011; Newton et al., 2012). In the United States, *V. vulnificus* is responsible for 95% of seafood-related deaths (Jones and Oliver, 2009). *V. cholerae*, the causative agent of cholera, is primarily non-saline fresh waterborne, and has been also associated with foods of terrestrial origin (Popovic et al., 1993). The illness produced by Vibrio bacteria is known as vibriosis and the symptoms include watery diarrhea, abdominal cramping, nausea, vomiting, fever, and chills (Baker-Austin et al., 2010). Usually the symptoms occur within 24 h of ingestion of the pathogen and last for about 3 days. Both *V. cholerae* and *V. parahaemolyticus* cause acute gastrointestinal symptoms, whereas *V. vulnificus* can cause serious and fatal infections like gastroenteritis, skin and soft tissue infections, and primary sepsis that may progress to necrotizing fasciitis and death if left untreated (Centers for Disease Control and Prevention, 2004; Bross et al., 2007).

## Diagnosis and Treatment of Food Borne Vibriosis

Laboratory diagnosis of *Vibrio* spp. includes enrichment of a sample of interest in alkaline peptone water, followed by culture on a selective media like thiosulfate–citrate–bile salts agar (TCBS) and/or CHROMagar Vibrio. Strains that are able to metabolize sucrose, such as *V. cholerae* will form yellow colonies on TCBS agar, whereas other pathogenic species like *V. parahaemolyticus* and *V. vulnificus* produce green colonies (Ramamurthy et al., 2020). On CHROMagar Vibrio, *V. parahaemolyticus* colony looks as mauve, whereas *V. cholerae* and *V. vulnificus* appears as turquoise (Passalacqua et al., 2016), then confirmed by a series of biochemical and serological tests to determine strain type. Molecular methods are currently available and widely used, including colony hybridization, polymerase chain reaction (PCR), real-time PCR, and loop-mediated isothermal amplification for species-level confirmation (Hill et al., 1991; Bej et al., 1999; Chun et al., 1999; Lyon, 2001; Campbell and Wright, 2003; Nordstrom et al., 2007). Recently culture-independent diagnostic tests (CIDT) are increasingly used to identify enteric pathogens including Vibrios that are commonly transmitted by food due to its better sensitivity than culture and shorter turnaround time (Langley et al., 2015; Shea et al., 2016; Tarr et al., 2019).

Treatment for *Vibrio* spp. infection mainly depends on the causative organism: for example, administration of oral or intravenous rehydration therapy for *V. cholerae* infection and surgical debridement of infected tissues for *V. vulnificus*-associated wound infections, with antibiotic therapy for severe cholera and systemic infections (Baker-Austin et al., 2018). Antibiotic therapy reduces the duration of diarrhea, severity of infection and limit further transmission of the disease (Lindenbaum et al., 1967). At present, azithromycin and ciprofloxacin are commonly used for *V. cholerae*, and doxycycline or quinolone for *V. parahaemolyticus*, whereas doxycycline and a third-generation cephalosporin are recommended for *V. vulnificus* as initial treatment regimen (Clemens et al., 2017; Baker-Austin et al., 2018). Ideally the choice of antibiotic should be based on antimicrobial susceptibility profiles of locally circulated pathogen/s.

## Emergence of Antimicrobial Resistance in Vibrios

*Vibrio* spp. are usually susceptible to commonly used antibiotics of human significance (Oliver, 2006). Earlier studies had shown that the strains of *V. vulnificus* were sensitive to tetracyclines, aminoglycosides, third-generation cephalosporins, chloramphenicol, and newer fluoroquinolones (Morris and Tenney, 1985; Tang et al., 2002; Baker-Austin et al., 2008). Similarly, *V. cholerae* was effectively treated with several antibiotics like tetracycline, azithromycin and fluoroquinolones over the years (Saha et al., 2006). But antibiotic resistance has emerged and evolved in many bacterial genera including Vibrios during the past few decades due to excessive use and misuse of antibiotics in human, agriculture, and aquaculture systems (Mazel and Davies, 1999; Cabello, 2006). Despite their public-health significance there are limited data on the effectiveness of antibiotic use in *V. vulnificus* and *V. parahaemolyticus* infections, in contrast to other enteric pathogens like *Salmonella* (Cabello, 2006; Han et al., 2007; Daniels, 2011). Recently, several studies have shown that *V. vulnificus* isolates became resistant to multiple antibiotics like ampicillin, tetracycline, aztreonam, streptomycin, gentamicin and tobramycin (Ji et al., 2011; Kim et al., 2011; Pan et al., 2012). Elmahdi et al. (2016) showed that most frequently observed antibiotic resistance for both *V. parahaemolyticus* and *V. vulnificus* is toward ampicillin, penicillin and tetracycline regardless of the countries. In a recent study, majority of *V. parahaemolyticus* isolates from different types of seafood in Malaysia were found to be susceptible to most antibiotics except ampicillin, cefazolin, and penicillin (Tan et al., 2020). Several other studies showed that majority of *V. parahaemolyticus* strains isolated from seafood, clinical, and environmental samples were highly resistant to multiple antibiotics like amoxicillin, ampicillin, bacitracin, carbenicillin, cefazolin, ceftazidime, cephalothin, colistin, gentamicin, penicillin, spectinomycin, and tobramycin (Zanetti et al., 2001; De Melo et al., 2011; Yano et al., 2011; Al-Othrubi et al., 2014; Elexson et al., 2014; Sudha et al., 2014).

Initially tetracycline, streptomycin and chloramphenicol were effectively used to treat cholera over the years (Reimann and Chang, 1946; Chaudhuri et al., 1950; Chakravarti et al., 1954; Carpenter et al., 1964; Uylangco et al., 1965; Saha et al., 2006). Recently, treatment failures are often observed with the recurrent emergence of antimicrobial resistant *V. cholerae* (Clemens et al., 2017). Unlike *V. vulnificus* and *V. parahaemolyticus*, more data is available on antimicrobial resistance of *V. cholerae.* Recently, Verma et al. (2019) carried out one of the largest analyses of antimicrobial resistance in *V. cholerae* strains (*n* = 443) isolated from the stool samples of diarrheal patients in India. In this study they selected 22 different antibiotics from nine different classes and showed that almost 99% of *V. cholerae* isolates were resistant against ≥2 antibiotics, 17.2% isolates were resistant against ≥10 antibiotics, and 7.5% isolates were resistant against ≥14 antibiotics. The highest resistance was detected against sulfamethoxazole (99.8%) and resistance to neomycin was observed to be lowest (4.0%). In addition, resistance to nalidixic acid, trimethoprim and streptomycin were also very high. Another study on antibiotic susceptibility pattern of *V. cholerae* was conducted from 2000 to 2018 in Bangladesh by Parvin et al. (2020) where they have shown that there was a rapid decline of the sensitivity of *V*. *cholerae* to tetracycline from nearly 100% to <6% during 2012–2017 and again increased to 76% in 2018. Susceptibility to azithromycin and ciprofloxacin was nearly 100% throughout the study period. Dengo-Baloi et al. (2017) performed antibiotic resistance profiling in *V. cholerae* (*n* = 159) that were isolated during cholera outbreaks in Mozambique from 2012 to 2015. They showed that all the isolates were resistant to ampicillin and nalidixic acid, 13% were also resistant to azithromycin and all of them were sensitive to ciprofloxacin.

Potential source of antibiotic resistance genes for Vibrios include horizontal gene transmission from other pathogens as well as commensal gut bacteria *via* various mobile genetic elements (Bag et al., 2019; Verma et al., 2019; Das et al., 2020). Antibiotic resistance genes are also acquired from the environment (Cantón, 2009) including aquatic bodies like lake also served as a potential reservoir of antibiotic resistance genes (Lepuschitz et al., 2019); effluents from wastewater treatment plants are also potential reservoirs of various antibiotics resistance genes (Okoh and Igbinosa, 2010).

## Mechanisms of Antimicrobial Resistance in Vibrios

**FIGURE 1:**
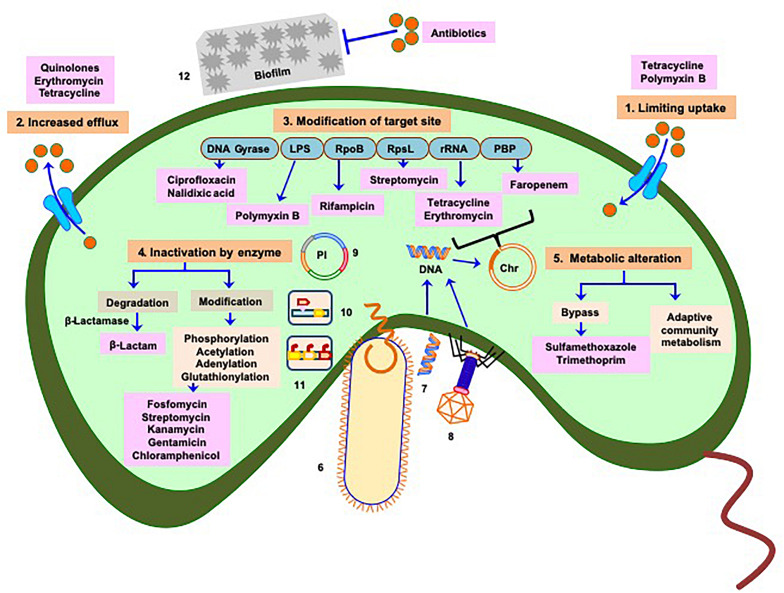
Manifestation of antibiotic resistance mechanisms of *Vibrio*: multiple mechanisms (intrinsic and acquired) responsible for acquisition of drug resistance or reduced susceptibility include 1. Limiting uptake of drug, 2. Increased drug efflux, 3. Modification of drug target site (chromosome associated phenomenon), 4. Inactivation of drug by enzymatic activity, and 5. Alteration of metabolism as depicted in saffron color boxes. Routes 6, 7, and 8 represent horizontal transfer of antibiotic resistance traits (mobile genetic elements) through conjugation, transformation, and phage transduction, respectively. Plasmids containing antibiotic resistance genes are referred in 9, 10, and 11 represent transfer of mobile genetic elements *via* transposons and integrons respectively. 12 Physicochemical phenomenon, such as formation of biofilm also play role in acquiring antibiotic resistance. Mechanisms depicted in 6–12 imply greater and faster probability of dissemination of resistance genes to clinical strains. Light blue boxes indicate targets of different antibiotics. Red balls denote concentration of antibiotics responsible for cellular influx or efflux by transporters (cyan) or efflux proteins (cyan), respectively. Antibiotics are represented in pink boxes. Pl represents plasmid, Chr represents chromosome. Based on the origin of antibiotic resistance it appears that most of the resistance mechanisms have been repurposed from intrinsic functions to the incorporation into Mobile Genetic Elements in pathogens.

Like other bacteria, the mechanisms of antibiotic resistance developed by Vibrios could be classified as (A) intrinsic (mutations originating within the organism) or (B) acquired through transfer of genetic elements during DNA replication (vertical transfer) or from different species or genera (horizontal transfer) (Figure 1).

### Intrinsic Resistance Mechanisms

These are natural conditions universally found in bacterial species and are independent of antibiotic selectivity (Cox and Wright, 2013). Some of them are as follows:

#### Limiting Uptake of a Drug Due to Permeability or Impermeability of the Outer Membrane or Cell Wall

Bacterial outer membrane forms a major permeability barrier. Changes in the expression of outer membrane porins (deduction, loss or replacement by structure alteration) can hinder entry of antibiotics (e.g., chloramphenicol, fluoroquinolones, tetracyclines, polymixin B, erythromycin, azithromycin, and rifamycin) (Delcour, 2009). Strains expressing full length Lipopolysaccharide (LPS) have an intrinsic resistance to hydrophobic antibiotic class, such as macrolides and aminoglycosides. Other common LPS modifications [cationic substitution of phosphate groups with 4-amino-4-deoxy-L-arabinose (L-Ara4N) or phosphoethanolamine (PEtN)] decrease the net negative charge of lipid A from −1.5 to −1 or from −1.5 to 0, respectively and reduce the binding of some cationic antibiotics, such as polymyxins, leading the resistance (Delcour, 2009). Modifications of the membrane lipid barrier can reduce the fluidity of the lipopolysaccharide (LPS) and also reduce permeability of antibiotics (carbapenems, tetracycline, fluoroquinolones, aminoglycosides, and chloramphenicol).

#### Active Drug Expulsion

The efflux pump proteins function by excluding out the antimicrobial compounds from the bacterial cell by utilizing energy from ATP or transmembrane ion gradients (Higgins, 2007). They can be encoded from chromosome or from extrachromosomal genetic elements (Sun et al., 2014). Some examples are (a) resistance-nodulation-cell division (**RND**) efflux protein (extrudes ampicillin, chloramphenicol, streptomycin and tetracycline), (b) major facilitator superfamily (**MFS**) (pumps out tetracycline), (c) Multidrug and toxic compound extrusion (**MATE**) families (expel compounds derived from β-lactams, macrolides, aminoglycosides, nalidixic acid, chloramphenicol SXT scaffolds), (d) ATP-binding cassette (**ABC**) protein (removes β-lactams, macrolides, aminoglycosides, nalidixic acid, tetracyclines, and SXT antibiotics), (e) Small multidrug resistance (**SMR**) family, etc.

#### Modification of the Target Site/Drug Target Modification

Point mutations in the target-encoding gene result into structural change of the target molecule which can alter antibiotic-target interactions limiting to antibiotic sensitivity (Andersson and Hughes, 2010). Examples include (a) *pbp* gene (cell wall biosynthesis affecting β-lactam), (b) *parC* and *gyrB* gene (DNA replication and repair affecting quinolone), (c) *rpsL* gene (protein synthesis affecting streptomycin), (d) *katG*, *embB*, *mshA* genes (metabolic enzymes affecting Sulfamethoxazole-trimethoprim- or SXT). Post-transcriptional modification, such as methylation of specific nucleotides in 16S or 23S rRNA by rRNA methyltransferase can also confer resistance against several aminoglycosides and macrolides antibiotics (Holmes et al., 2016).

#### Inactivation of a Drug

Vibrios can either destroy antibiotic function by enzymes hydrolyzing the core drug structure or by chemical modification through transferring a chemical group to the scaffolds (Pascale and Wright, 2010). For example metallo-β-lactamase (class B) or serine-β-lactamase (classes A, C, and D) destroys β-lactams (Bush and Bradford, 2016). Erythromycin esterases EreA and EreB hydrolyze the macro lactone rings of macrolides, such as erythromycin except telithromycin (semisynthetic erythromycin derivative) (DrugBank database. Telithromycin, 2019. Available from: https://www.drugbank.ca/drugs/DB00976; Accessed: 12 January 2021). Enzymatic modification of antibiotic includes (a) *O-phosphorylation* (fosfomycin), (b) *O-nucleotidylylation* [nucleotidyl transferases (ANT)] (several aminoglycosides), (c) *O-glycosylation* (macrolide and rifampin), (d) *O- and N-acetylation* (Chloramphenicol, fluoroquinolone, streptothricin, and other aminoglycosides), (e) *O-ribosylation*, (f) *hydroxylation*, etc. (D’Costa and Wright, 2009).

#### Alternative Metabolic Pathway

Sulfamethoxazole and trimethoprim inhibit bacterial folate synthesis pathway, but the acquisition of *sul2* and *dfrA1* genes renders sulfamethoxazole and trimethoprim resistance, respectively. The physiology engendered tolerance, or decreased susceptibility can occur due to innate cellular property or regulatory circuit present in the organism. For example, a change in nutrient pools within a polymicrobial community may induce a change in the inherent susceptibility of certain organisms to a given antibiotic mediated by alteration in bacterial metabolism (Holmes et al., 2016).

However, based on the origin of antibiotic resistance it appears that most of the resistance mechanisms have been repurposed from intrinsic functions to the incorporation into Mobile Genetic Elements in pathogens that poses a considerable threat to human health.

### Acquired Resistance Mechanisms

In the spectrum of antibiotic resistance mechanisms spontaneous mutation (appear from non-synonymous point mutation or form of insertion elements) inherited by the daughter cells through vertical transfer is relatively a slow process (Figure 1) that can modify target enzymes, alter transcription of select genes, or bypass antibiotic activity (Wyk, 2015). Recent studies showed that genetic basis of the emergence of multidrug resistant (MDR) and extensively drug resistant (ExDR) Vibrios and other enteric pathogenic bacteria is mainly due to horizontal gene transfer (HGT) through different types of highly dynamic mobile genetic elements, such as plasmids, integrating conjugative elements, superintegron, transposable elements, and insertion sequences (Wozniak and Waldor, 2010; Kumar et al., 2017; Verma et al., 2019) that could potentially propagate from one bacteria to other closely or distantly related bacteria. Natural competency in Vibrios allows them to uptake and chromosomally integrate, exogenous DNA coming from multidrug resistant commensal gut bacteria and other sources which ultimately leads to the emergence of drug resistant Vibrios (Meibom et al., 2005; Bag et al., 2019; Sinha-Ray et al., 2019). A novel “carry-back” mechanism for inter-phylum exchange of genes is also proposed where carrier DNA sequence may be transferred from *Proteobacteria* to *Actinobacteria via* conjugative plasmid or integron and then again taken up by *Proteobacteria* from *Actinobacteria* through transformation followed by genomic integration through homologous recombination. This is then easily transferred to pathogenic bacteria which also mostly belong to the phylum Proteobacteria (Jiang et al., 2017). HGT is encouraged by the density of microbial communities and can also introduce multiple fitness factors even in the single event of acquisition. Acquiring and shedding of genetic traits encoding resistance often results more rapidly than spontaneous mutations (Wozniak and Waldor, 2010).

Bacteria can also respond to antibiotics through phenotypic variation, metabolic fluxes, programmed responses to the environment, or a systems-level effect of the bacterial community (Corona and Martinez, 2013). Enrichment of antibiotic resistance certainly depends on selective pressure, fitness cost, and dispersal (Bengtsson-Palme et al., 2018). Certain environmental hot-spot, such as sewage and wastewater treatment plants, hospital effluents, aquaculture, agricultural and slaughterhouse waste, are prime locations for genetic exchange events because of the high density of bacteria, phages, and plasmids in these regions (von Wintersdorff et al., 2016). Physicochemical phenomenon, such as formation of biofilm and quorum sensing also play the role. Biofilm formation which consists of cells, debris, proteins, polysaccharides, and extracellular DNA provides a protective covering to the bacterial community by helping the bacteria secrete enzymes which degrade antibiotics, and other antimicrobials (Singh et al., 2017). Bacterial quorum sensing induces the expression of biofilm formation. It activates several signaling pathways that cause autolysis of the cells present in the biofilm, and the extracellular DNA helps the bacteria thriving against the antibiotics (Abisado et al., 2018). Sometimes these adaptive strategies may not result in absolute resistance, they may promote tolerance in the population and allow time for the organisms to acquire extensive AMR through other mechanisms.

Primary modes of transmission of antibiotic resistance genes between different Vibrios and other bacteria are HGT pathways including conjugation, transduction, transformation and fusion (Davies and Davies, 2010; Soucy et al., 2015; Bag et al., 2019). HGT plays an important role in the acquisition of antibiotic resistance in bacteria including Vibrios. Comprehensive whole genome sequencing studies suggest that drug-resistant Vibrios harbor mobile genetic elements like plasmids, integrating conjugative elements e.g., SXT, gene cassettes, and integrons (Beaber et al., 2002; Rapa and Labbate, 2013; Partridge et al., 2018; Das et al., 2020). Interestingly, Tox R expression (by *tox R* gene) in food borne *V. parahaemolyticus* is able to regulate the production of thermostable direct hemolysin (TDH) (*tdh* gene), TDH related hemolysin (TRH) (*trh* gene), Type III Secretion Systems T3SS1 and T3SS2 which are intrinsic factors that can cause damage from a distance even in absence of direct pathogens and hence remain ineffective in presence of antibiotics (Hubbard et al., 2016; Mala et al., 2016; Mariya Sony et al., 2021). Some clinical *V. parahaemolyticus* strains, however, remains pathogenic without having the mentioned virulence factors indicating the existence of putative factors and pathogenicity might be achieved with different strategies employed by different strains (Cai and Zhang, 2018). Recent study identified high prevalence (>70%) of hemolysin *vhh*, the quorum-sensing regulator *luxR*, chitinase *chiA*, and the transmembrane transcription regulator *toxR*_*Vh*_ in the typical host *V. harveyi* and *flaC* in *V. anguillarum*, *vhh* in *V. vulnificus* in South China (Deng et al., 2020). Presence of *tetB/tetH* gene was identified as the predictor for the resistance against the first generation tetracycline, the most commonly used antibiotic against *Vibrio* spp. in marine and brackish aquaculture systems of India (Mariya Sony et al., 2021). In addition to antibiotic abuse, warming temperatures, acid–base and organic pollution can directly induce the expression of such antibiotic resistance genes and virulence genes and also affect HGT (Guijarro et al., 2015; Deng et al., 2020). A precise and critical view has been included in Table 1 which summarizes the presence of different antibiotic resistance genes identified in several *Vibrio* spp. including *V. parahaemolyticus*, *V. cholerae*, *and V. vulnificus* by various antibiotic resistance determinants specific PCR amplifications and/or whole genome sequencing.

**TABLE 1:** Presence of antibiotic resistance genes in different *Vibrio* spp.

***Vibrio* spp.**	**Isolated from**	**Antibiotic resistance genes**	**References**
*V. parahaemolyticus*	Shrimps	class 1 integrase, *sul*2, *strB*, and *catB*3	Beshiru et al., 2020
	Oysters	*qnr*, *str*B, *tet*(A), *bla*TEM	Jeamsripong et al., 2020
	Fish	*strB*, *blaP1*, *floR*, *tetA*, *ermB*, *qnrA*, *aac*(3)-*IIa*	Faja et al., 2019
*V. cholerae*	Diarrheal patients	*dfFrA1*, *sul1*, *mphR*, *mrx*, *mphA*, *dfrA15*, *merRTP*, *FADE*	Morita et al., 2020
		*bla(A)*, *bla(D)*, *ant(3’)*, *aac(3’),sh ble*, *bla_*NDM*_(B)*, *aph(3’)*, *cat*, *marR*, SXT int, *strA*, *strB*, *tehA*, *acrA*, *vexC*	Verma et al., 2019
	Stool of cholera patient	*gyr*A (Ser83Ile), *par*C (Ser85Leu), *sul*2, *str*A/*str*B, *flo*R, *cat*B9, *dfr*A1	Kaas et al., 2016
*V. vulnificus*	Recreational beaches	*bla*_*NDM*__–__1_, *bla*_*TEM*_, *bla*_*CMY*_	Oyelade et al., 2018
	Fish cultures	SXT int, *strB*, *tetA*, *sul2*	García-Aljaro et al., 2014

## Lack of New Antibiotics and Alternative Antibiotic Therapy

Lack of new antibiotics threatens global efforts to contain drug-resistant infections. There are considerable delay in the development of new class of antibiotics from the past decades. Two new classes of antimicrobials (oxazolidinones and lipopeptides) (Prokocimer et al., 2012) developed against MDR pathogens are not very efficient against Gram-negative bacteria like Vibrios (Hughes and Karlen, 2014). Other antibiotics developed are of existing classes only and 9 out of 109 candidates could only reach into phase III trials in 2012 (Hughes and Karlen, 2014). However, problems associated with antibiotic resistance are constantly evolving. Hence, cutting age techniques including adaptive laboratory evolution with antibiotic stress followed by whole-genome sequencing need frequent use to understand the evolution in real time. Apart from phenotypic characterization culture-independent metagenome and resistome analysis could provide better picture of the genetic potential of bacteria against new classes of antibiotics.

Alternative therapy using therapeutic phages targeting the pathogenic bacteria are much safer than antibiotics. Natural compounds (antimicrobial peptides from frogs, alligators, and cobras, phytochemicals, polyphenols, essential oils from plants) having antibacterial property and identification of compounds acting against bacterial toxins rather than killing the bacteria as such would be the promising approaches (Yaraksa et al., 2014; Upadhyay et al., 2015; Macwan et al., 2016). High-throughput *in silico* analysis to identify novel compound/s and their mode of action on *Vibrios* are highly warranted. Application of non-pathogenic bacteria from the natural flora (probiotic therapy) (*Bdellovibrio bacteriovorus*, *Micavibrio aeruginosavorus*, *Ruminococcus obeum*) have been found to attenuate the disease progression (Dwidar et al., 2012). Several antivirulent strategies, such as targeting bacterial adhesion, colonization, toxin production, and quorum sensing have been developed recently by chemical compounds, and natural compounds, such as phytochemicals and nanotechnology. Examples are Virstatin (4-[N-(1,8-naphthalimide)]-n-butyric acid) (block ToxT gene); 6- Gingerol and Zinc oxide nanoparticles (prevent interaction of cholera toxin with GM1 receptor); Lactoferrin in milk has antiadhesive activity by iron chelation (Ammons and Copié, 2013). Many metal oxides, polymer nanoparticles, metal nanoparticle-DNA aptamer conjugates, Flash NanoPrecipitation of water-dispersible CAI-1 autoinducer nanocarriers have been identified to have potent antibiofilm activity. Other strategies, such as oral vaccines [Dukoral (SBL Vaccines), Shanchol (Shantha Biotech, India), Euvichol (EuBiologic Co., Ltd., Chuncheon, South Korea), and mORCVAX (Vabiotech, Hanoi, Vietnam)] are widely used against cholera to confer moderate protection, but, herd immunity can multiply the effectiveness (Martin et al., 2014). Vaccines in combination with other preventive measures, such as regulation of needless use of antibiotics, WASH (Water, sanitation, and hygiene) program, proper cooking of foods (especially seafoods), keeping food away from contaminants, washing fruits and vegetables before cooking may help in reducing the antimicrobial use and thereby decrease the chances of developing AMR. Communal participation to tackle the challenge of AMR is also very critical.

## Future Directions and Concluding Remarks

Vibrios, the autochthonous inhabitant of aquatic environment, upon infection seeded into the environment where it acquires and transmits new genes, including antibiotic resistance. To prevent and control the spread of food borne vibriosis, the consumers should avoid eating raw or undercooked seafoods as they are the significant source of potentially pathogenic Vibrios. Several decontamination methods like high pressure processing technique and the use of different effective chemicals are available to effectively reduce the number of Vibrios without compromising the food-value of raw and finished food products (Mata et al., 1994; de Castillo et al., 1998; Berlin et al., 1999; Cook, 2003).

Misuse and overuse of antibiotics leads to the emergence of antimicrobial resistance in microorganisms including Vibrios and it is increasing across the globe that poses a global threat to public health. It requires urgent multisectoral action in order to achieve the goals of sustainable development. Because of widespread resistance to antibiotics, robust surveillance is needed to detect changing sensitivity patterns for determining the drug of choice for treating Vibrio infections. It is crucial to determine the mechanisms of virulence and the consequences of host–pathogen interactions from both the pathogen and host perspective. With cutting age techniques identification of bacterial virulence along with knowledge of genomes should be used to identify novel targets to design and develop multidisciplinary and novel treatments.

All countries should actively participate in the Global Antimicrobial Resistance Surveillance System (GLASS) which was launched by the World Health Organization in 2015 to support the global action plan on antimicrobial resistance (World Health Organization, 2015). Although several programs have been implemented in countries, such as EU, United States, Japan, Sweden, and Denmark and recently in countries, such as India, China, Thailand, and South Africa (Founou et al., 2016), a consolidated action plan is warranted to take proactive steps toward prevention of food borne pathogen’s spread (Centers for Disease Control and Prevention, 2018).

To tackle the infections caused by multidrug resistant and extensive drug resistant Vibrios and other bacteria there is an urgent need to develop new antibiotics or modern-day alternative therapeutics but unfortunately this has slowed considerably in past decades. During this health crisis, the infections can be prevented if we all have better hygiene, access to clean water and sanitation, infection control in healthcare facilities and vaccination, which could help us to reduce the need for antibiotics. In the age of multi-drug resistance, the universal decline in the effectiveness of antibiotics has generated renewed interest in revisiting the practice of phage therapy by cocktail treatment (Jun et al., 2014), usage of quorum sensing inhibitors, and anti-secretory drugs and more research efforts are essential on probiotic therapy (Hsiao et al., 2014) and re-sensitization of drug resistant pathogens (Sun et al., 2020).

## Author Contributions

DD and SB conceptualized and wrote the manuscript. AK and DK edited the review. All authors read and approved the review.

## Conflict of Interest

SB was employed by company: 3B BlackBio Biotech India Limited. The remaining authors declare that the research was conducted in the absence of any commercial or financial relationships that could be construed as a potential conflict of interest.
